# Soft skills in Orthodontics: an analysis in residents and experienced professionals

**DOI:** 10.1590/2177-6709.29.2.e242370.oar

**Published:** 2024-06-10

**Authors:** Ivan SILVA, Felicia MIRANDA, José Roberto Pereira LAURIS, Daniela GARIB

**Affiliations:** 1 University of São Paulo, Bauru Dental School, Department of Orthodontics (Bauru/SP, Brazil).; 2 University of São Paulo, Hospital for Rehabilitation of Craniofacial Anomalies (Bauru/SP, Brazil).; 3 University of São Paulo, Bauru Dental School, Department of Public Health (Bauru/SP, Brazil).

**Keywords:** Orthodontics, Social skills, Work performance, Ortodontia, Habilidades sociais, Desempenho no trabalho

## Abstract

**Introduction:**

Soft skills represent a range of personal skills, attitudes and characteristics relevant to success and adequate work performance.

**Objective:**

This study aimed to evaluate the knowledge and usage of soft skills in Orthodontics.

**Methods:**

The participants answered a questionnaire containing 27 objective questions on awareness and frequency of soft skills in their professional activities. Participants were also asked to rank the soft skills in a crescent order of importance. The sample was divided into subgroups: A) residents in Orthodontics; B) orthodontists with less than 5 years of experience and C) orthodontists with more than 5 years of experience. Intergroup comparisons were performed using the Kruskal-Wallis test. Sexual differences were compared using Mann-Whitney test (*p*<* *0.05).

**Results:**

The sample of this observational study comprised 129 experienced orthodontists and residents in Orthodontics (92 women, 37 men) with mean age of 35.3 years. From the total sample, 54,6% of respondents reported no previous instructions on soft skills. All respondents reported using the analyzed soft skills with a similar frequency (median 4-5). Residents reported accessing reliable sources in bibliographic research less frequently (46%). Female orthodontists reported to seek help from teachers and other professionals more often than males. Ethics and communication were frequently ranked as the most important soft skills. Information management and leadership were frequently less ranked as important soft skills.

**Conclusion:**

Poor knowledge of soft skills was demonstrated by residents and orthodontists. Communication skill was highly used and frequently ranked as the most important soft skill.

## RESUMO

**Introdução:**

As *soft skills* representam uma variedade de habilidades pessoais, atitudes e características relevantes para o sucesso e desempenho adequado no trabalho.

**Objetivo:**

Este estudo teve como objetivo avaliar o conhecimento e o uso das *soft skills* em ortodontistas.

**Métodos:**

Os participantes responderam a um questionário contendo 27 perguntas objetivas sobre conhecimento e frequência das *soft skills* em suas atividades profissionais. Os participantes também foram solicitados a classificar as *soft skills* em uma ordem crescente de importância. A amostra foi dividida em subgrupos: A) residentes em Ortodontia; B) ortodontistas com menos de 5 anos de experiência e C) ortodontistas com mais de 5 anos de experiência. As comparações intergrupos foram realizadas usando o teste de Kruskal-Wallis. Diferenças entre os sexos foram comparadas usando o teste de Mann-Whitney (*p*< 0,05).

**Resultados:**

A amostra deste estudo observacional compreendeu 129 ortodontistas experientes e residentes em Ortodontia (92 mulheres, 37 homens) com idade média de 35,3 anos. Entre os entrevistados, 54,6% relataram não ter recebido instruções prévias sobre as *soft skills*. Todos os entrevistados relataram usar as habilidades interpessoais analisadas com uma frequência semelhante (mediana 4-5). Os residentes relataram acessar com menos frequência (46%) fontes confiáveis de pesquisa bibliográfica. Ortodontistas do sexo feminino relataram buscar ajuda de professores e outros profissionais com mais frequência do que os do sexo masculino. Ética e comunicação foram frequentemente classificadas como as *soft skills* mais importantes. Com frequência, o gerenciamento de informações e a liderança foram classificados como *soft skills* menos importantes.

**Conclusão:**

Foi demonstrado pouco conhecimento sobre as *soft skills* por parte de residentes e ortodontistas. A habilidade de comunicação foi frequentemente utilizada e considerada a *soft skill* mais importante.

## INTRODUCTION

Globalization has changed the references of interaction and relationship patterns between people.^1^ Health professionals should be trained not only in technical and scientific skills, but also in social skills.^1^ The educational institutions in Dentistry already grant their students great cognitive and psychomotor attributes. However, there has been a worldwide educational movement that values ​​instructions of non-cognitive tools related to emotional intelligence.^2^

Soft skills are a range of personal skills, characteristics, and attitudes that represent key factors for success at work and in academic environments.^3^ Soft skills are related to better professional performance and can be improved with training.^4^ In Dentistry, characteristics such as communication, professionalism and good coordination of the work team increase patient reliability in the professional work, and increase the success of clinical practices.^5^ The main soft skills addressed in higher education programs in Dentistry are communication, critical thinking, teamwork, leadership and professionalism.^6^

Recent studies reveal that soft skills have been addressed within Dentistry education around the globe.^3^ ^,^ ^7^ ^,^ ^8^ UK researchers defend assessing soft skills and sensorimotor skills as part of the selection for students who intend to be dentists.^7^ Dentistry residents from Pakistan have recognized the importance of soft skills in their profession, even though leadership was not considered important.^8^ Undergraduate students from Malaysia considered teamwork and communication the most relevant skills.^9^ In Iran, the soft skills considered relevant by undergraduate students were professional ethics, artistic skills (manual skill and esthetic vision) and cognitive skills (creativity, critical sense and decision-making).^3^ From a work perspective, the use of non-technical skills is associated with greater employability and better clinical outcomes.^9^

No previous study has evaluated the influence of soft skills in the orthodontic field. This study aimed to assess the knowledge, usage, and importance priority of soft skills by both orthodontists and residents in Orthodontics.

## METHODS

This study was approved by the Ethics in Research Committee of São Paulo University, Bauru Dental School (Brazil, under protocol no. 53485621.8.0000.5417). All participants signed an informed consent. 

**Figure 1:**
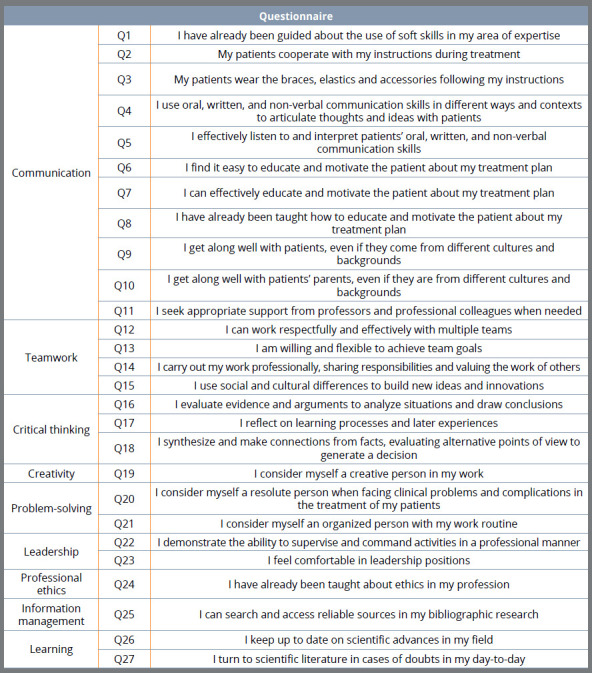
Questionnaire.

Volunteers, represented by orthodontists and residents in orthodontics, were invited to answer an online questionnaire with 34 questions using Google Forms. The questionnaire (Fig 1) explored nine soft skills relevant to Dentistry, according to the scientific literature.^3^ ^,^ ^8^ The soft skills included were communication, teamwork, critical thinking, problem-solving, creativity, leadership, professional ethics, learning and information management. 

In the first part of the questionnaire, through a Likert scale (never, rarely, sometimes, almost always, always), participants answered the frequency that they performed or were aware of soft skills in professional activities. Secondly, respondents were asked to rank the importance of nine soft skills. The participants ranked the soft skills from one to nine, according to their personal order of importance, in which one was the least important and nine, the most important. 

The sample calculation for this study was based on the sample size of a previous cross-sectional research^8^ that aimed to develop a soft skills questionnaire for 60 Dentistry students. The Google forms were sent to orthodontists and residents in orthodontics, using WhatsApp. The contact of the dentists was collected through a list of 1 688 professionals and residents from the Brazilian Association of Orthodontics. 

The final sample of this observational study comprised 129 experienced orthodontists and residents in Orthodontics (92 women, 37 men) with a mean age of 35.3 years. This research demonstrated a response rate of 7.6%. The sample was divided into subgroups: A) residents in orthodontics (n=39); B) orthodontists with less than 5 years of experience (n=37) and C) orthodontists with more than 5 years of experience (n=53). 

## STATISTICAL ANALYSIS

The frequencies of the Likert scale were converted into a numerical order, from one (never) to five (always), for statistical purposes. In the ranking process, the sum of all the numbers selected by the participants for each soft skill was calculated. The skills with the lowest total value were the less important, while the ones that presented the highest values in the total sum were considered more important. 

Intergroup comparison was performed using Kruskal-Wallis tests. A *post-hoc* pairwise comparison was also performed in case of statistical difference. Mann-Whitney U test was used to compare sexes. Data analysis was carried out using the JAMOVI (version 1.2) open-source statistical software (https://www.jamovi.org). Results were considered significant at *p*<0.05.

## RESULTS

Descriptive statistics of the sample are described in Table 1. The absolute and relative frequencies of all groups are described in Table 2. Most respondents (54,6%) reported that never received instructions regarding the use of soft skills during their training (Table 2). Respondents also frequently reported getting along well with patients and their parents with high frequency (average of 64% and 57%, respectively). In addition, acting professionally with responsibility and flexibility with teamwork were frequently reported (average of 72%, 73% and 81%).

**Table 1:** Descriptive statistic.

	Mean	Median	S.D.
Q1	1.91	1	1.2
Q2	3.83	4	0.42
Q3	3.92	4	0.37
Q4	4.25	4	0.85
Q5	4.22	4	0.7
Q6	3.98	4	0.67
Q7	3.86	4	0.55
Q8	2.98	3	1.1
Q9	4.6	5	0.59
Q10	4.52	5	0.6
Q11	4.13	4	1.01
Q12	4.71	5	0.49
Q13	4.72	5	0.48
Q14	4.78	5	0.47
Q15	3.85	4	1.03
Q16	4.33	4	0.7
Q17	4.4	5	0.73
Q18	4.13	4	0.77
Q19	3.84	4	0.85
Q20	4.19	4	0.67
Q21	4.19	4	0.83
Q22	4.12	4	0.81
Q23	3.79	4	1.07
Q24	4.26	5	0.94
Q25	4.49	5	0.73
Q26	4.36	4	0.69
Q27	4.15	4	0.87

**Table 2:** Absolute and relative frequencies.

	Group A (n= 39)					Group B (n= 37)					Group C (n=53)				
	Never	Rarely	Sometimes	Almost always	Always	Never	Rarely	Sometimes	Almost always	Always	Never	Rarely	Sometimes	Almost always	Always
Q1	22 (56%)	9 (23%)	5 (12%)	3 (7%)	0	20 (54%)	4 (10%)	7 (18%)	4 (10%)	2 (5%)	29 (54%)	10 (18%)	6 (11%)	4 (7%)	4 (7%)
Q2	0	0	9 (23%)	30 (76%)	0	0	0	5 (13%)	31 (83%)	1 (2%)	0	0	9 (16%)	43 (81%)	1 (2%)
Q3	0	0	3 (7%)	34 (87%)	2 (5%)	0	0	6 (16%)	30 (81%)	1 (2%)	0	0	6 (11%)	46 (86%)	1 (2%)
Q4	0	1 (2%)	10 (25%)	13 (33%)	15 (38%)	0	0	5 (13%)	16 (43%)	16 (43%)	1 (2%)	2 (4%)	5 (9%)	15 (28%)	30 (56%)
Q5	0	0	4 (10%)	22 (56%)	13 (33%)	0	1 (2%)	3 (8%)	18 (48%)	15 (40%)	1 (2%)	0	5 (9%)	30 (56%)	17 (32%)
Q6	0	1 (2%)	8 (20%)	24 (61%)	6 (15%)	0	0	8 (21%)	23 (62%)	6 (16%)	0	1 (2%)	8 (15%)	30 (56%)	14 (26%)
Q7	0	0	12 (30%)	23 (58%)	4 (10%)	0	0	10 (27%)	25 (67%)	2 (5%)	0	0	8 (15%)	39 (73%)	6 (11%)
Q8	3 (7%)	6 (15%)	15 (38%)	11(28%)	4 (10%)	1 (2%)	9 (24%)	13 (35%)	10 (27%)	4 (10%)	9 (16%)	15 (28%)	14 (26%)	13 (24%)	2 (4%)
Q9	0	0	3 (7%)	12 (30%)	24 (61%)	0	1 (2%)	1 (2%)	11 (29%)	24 (64%)	0	0	0	17 (32%)	36 (67%)
Q10	0	0	4 (10%)	14 (35%)	21 (53%)	0	0	3 (8%)	12 (32%)	22 (59%)	0	0	0	22 (41%)	31 (58%)
Q11	0	2 (5%)	4 (10%)	11(28%)	22 (56%)	0	1 (2%)	9 (24%)	7 (18%)	20 (54%)	0	9 (16%)	9 (16%)	14 (26%)	21 (39%)
Q12	0	0	1 (2%)	7 (17%)	31 (79%)	0	0	0	11 (29%)	26 (70%)	0	0	1 (2%)	16 (30%)	36 (67%)
Q13	0	0	1 (2%)	7 (17%)	31 (79%)	0	0	0	9 (24%)	28 (75%)	0	0	1 (2%)	16 (30%)	36 (67%)
Q14	0	0	1 (2%)	3 (7%)	35 (89%)	0	0	1 (2%)	5 (13%)	31 (83%)	0	0	1 (2%)	14 (26%)	38 (71%)
Q15	0	5 (12%)	9 (23%)	11 (28%)	14 (35%)	0	3 (8%)	9 (24%)	16 (43%)	9 (24%)	4 (7%)	0	13 (24%)	19 (35%)	17 (32%)
Q16	0	2 (5%)	4 (10%)	19 (48%)	14 (35%)	0	0	4 (10%)	16 (43%)	17 (45%)	0	0	3 (5%)	23 (43%)	27 (50%)
Q17	0	2 (5%)	3 (7%)	19 (48%)	15 (38%)	0	1 (2%)	2 (5%)	11 (29%)	23 (43%)	0	0	5 (9%)	19 (35%)	29 (54%)
Q18	0	2 (5%)	8 (20%)	17 (43%)	12 (30%)	0	2 (5%)	5 (13%)	20 (54%)	10 (27%)	0	0	6 (11%)	25 (47%)	22 (41%)
Q19	1 (2%)	5 (12%)	10 (25%)	16 (41%)	7 (17%)	0	0	10 (27%)	19 (51%)	8 (21%)	0	1 (2%)	15 (28%)	23 (43%)	14 (26%)
Q20	0	3 (7%)	3 (7%)	20 (51%)	13 (33%)	0	0	1 (2%)	27 (72%)	9 (24%)	0	0	6 (11%)	28 (52%)	19 (35%)
Q21	0	4 (10%)	5 (12%)	13 (33%)	17 (43%)	0	1 (2%)	2 (5%)	20 (54%)	14 (37%)	0	2 (4%)	6 (11%)	24 (45%)	21 (39%)
Q22	0	3 (7%)	8 (20%)	16 (41%)	12 (30%)	0	1 (2%)	4 (10%)	15 (40%)	17 (45%)	0	0	11 (20%)	24 (45%)	18 (33%)
Q23	3 (7%)	3 (7%)	10 (25%)	17 (43%)	6 (15%)	1 (2%)	5 (13%)	5 (13%)	14 (37%)	12 (32%)	2 (4%)	2 (4%)	9 (16%)	23 (43%)	17 (32%)
Q24	0	1 (2%)	8 (20%)	8 (20%)	22 (56%)	0	3 (8%)	3 (8%)	10 (27%)	21 (56%)	1 (2%)	3 (5%)	7 (13%)	16 (30%)	26 (49%)
Q25	0	0	5 (12%)	16 (41%)	18 (46%)	0	1 (2%)	2 (5%)	5 (13%)	29 (78%)	0	0	8 (15%)	12 (22%)	33 (62%)
Q26	0	1 (2%)	6 (15%)	19 (48%)	13 (33%)	0	0	3 (8%)	16 (43%)	18 (48%)	0	0	4 (7%)	19 (35%)	30 (56%)
Q27	0	3 (7%)	9 (23%)	13 (33%)	14 (35%)	0	1 (2%)	8 (21%)	13 (35%)	15 (40%)	0	1 (2%)	9 (16%)	17 (32%)	26 (49%)

Table 3 presents the intergroup comparison. The three subgroups showed similar scores for all questions, except for question #25, on searching and accessing reliable sources (*p* = 0.035). Orthodontists with less than five years of experience were more frequently able to search and access reliable sources of information (78%), when compared to residents (46%).

**Table 3:** Intergroup comparison (Kruskal-Wallis test).

	Group A (n= 39)			Group B (n= 37)			Group C (n=53)			P-value*
	Mean	S.D.	Median	Mean	S.D.	Median	Mean	S.D.	Median	
Q1	1.72	0.97	1	2.05	1.31	1	1.94	1.29	1	0.711
Q2	3.77	0.42	4	3.86	0.41	4	3.85	0.41	4	0.569
Q3	3.97	0.36	4	3.89	0.39	4	3.91	0.35	4	0.569
Q4	4.08	0.87	4	4.30	0.70	4	4.34	0.93	5	0.187
Q5	4.23	0.62	4	4.27	0.73	4	4.17	0.75	4	0.774
Q6	3.90	0.68	4	3.95	0.62	4	4.08	0.70	4	0.378
Q7	3.79	0.61	4	3.78	0.53	4	3.96	0.51	4	0.206
Q8	3.18	1.07	3	3.19	1.02	3	2.70	1.14	3	0.066
Q9	4.54	0.64	5	4.57	0.68	5	4.68	0.47	5	0.685
Q10	4.44	0.68	5	4.51	0.65	5	4.58	0.49	5	0.708
Q11	4.36	0.87	5	4.24	0.92	5	3.89	1.12	4	0.095
Q12	4.77	0.48	5	4.70	0.46	5	4.66	0.51	5	0.490
Q13	4.77	0.48	5	4.76	0.43	5	4.66	0.51	5	0.452
Q14	4.87	0.40	5	4.81	0.46	5	4.70	0.50	5	0.098
Q15	3.87	1.06	4	3.84	0.89	4	3.85	1.12	4	0.912
Q16	4.15	0.81	4	4.35	0.67	4	4.45	0.60	5	0.219
Q17	4.21	0.80	4	4.51	0.73	5	4.45	0.66	5	0.119
Q18	4.00	0.85	4	4.03	0.79	4	4.30	0.66	4	0.162
Q19	3.59	1.02	4	3.95	0.70	4	3.94	0.79	4	0.231
Q20	4.10	0.85	4	4.22	0.47	4	4.25	0.64	4	0.846
Q21	4.10	0.99	4	4.27	0.69	4	4.21	0.79	4	0.936
Q22	3.95	0.91	4	4.30	0.77	4	4.13	0.73	4	0.205
Q23	3.51	1.10	4	3.84	1.12	4	3.96	0.99	4	0.104
Q24	4.31	0.89	5	4.32	0.94	5	4.19	1.00	4	0.750
Q25	4.33^A^	0.70	4	4.68^B^	0.70	5	4.47^AB^	0.74	5	0.035*
Q26	4.13	0.76	4	4.41	0.64	4	4.49	0.63	5	0.054
Q27	3.97	0.95	4	4.14	0.85	4	4.28	0.81	4	0.295

*Statistically significant (*p* < 0.05). Different superscript letters indicate statistically significant differences.

Both men and women showed similar findings, except for question #11 (*p* = 0.005). Women demonstrated seeking support from professors and colleagues more often than men (Table 4).

**Table 4:** Comparison of answers between the sexes (Mann-Whitney U test).

Questions	Male (n= 37)			Female (n= 92)			p-value*
	Mean	S.D.	Median	Mean	S.D.	Median	
Q1	2.0	1.2	2	1.9	1.2	1	0.488
Q2	3.8	0.4	4	3.9	0.4	4	0.220
Q3	3.9	0.4	4	3.9	0.4	4	0.952
Q4	4.2	0.9	4	4.3	0.9	4	0.739
Q5	4.1	0.8	4	4.3	0.7	4	0.488
Q6	3.9	0.7	4	4.0	0.6	4	0.745
Q7	3.9	0.6	4	3.8	0.6	4	0.693
Q8	2.9	1.1	3	3.0	1.1	3	0.528
Q9	4.5	0.7	5	4.6	0.5	5	0.610
Q10	4.5	0.6	5	4.5	0.6	5	0.658
Q11	3.8	1.1	4	4.3	1.0	5	0.005*
Q12	4.6	0.5	5	4.7	0.5	5	0.288
Q13	4.7	0.5	5	4.7	0.5	5	0.558
Q14	4.8	0.4	5	4.8	0.5	5	0.430
Q15	3.8	1.2	4	3.9	1.0	4	0.836
Q16	4.4	0.7	4	4.3	0.7	4	0.674
Q17	4.3	0.7	4	4.4	0.7	5	0.426
Q18	4.2	0.7	4	4.1	0.8	4	0.626
Q19	4.0	0.8	4	3.8	0.9	4	0.190
Q20	4.2	0.6	4	4.2	0.7	4	0.965
Q21	4.2	0.8	4	4.2	0.9	4	0.560
Q22	4.3	0.7	4	4.1	0.8	4	0.265
Q23	4.0	1.0	4	3.7	1.1	4	0.070
Q24	4.2	0.8	4	4.3	1.0	5	0.244
Q25	4.6	0.6	5	4.4	0.8	5	0.199
Q26	4.5	0.6	5	4.3	0.7	4	0.080
Q27	4.2	0.8	4	4.1	0.9	4	0.766

*Statistically significant at *p* < 0.05.

**Figure 2:**
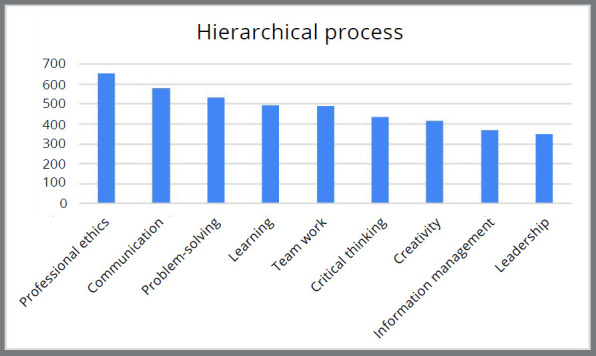
Hierarchical process.

Professional ethics and communication were most frequently ranked as important soft skills (Fig 2). Information management and leadership were less frequently considered important soft skills (Fig 2). The following descending order of importance was found for the nine skills: professional ethics, communication, problem-solving, learning, teamwork, critical thinking, creativity, information management and leadership. 

## DISCUSSION

This is the first study to investigate the use and application of soft skills in Orthodontics. Soft skills are subjective abilities that act in the behavioral and social spectrum of human beings.^5^ The enhancement of emotional abilities has been described as an important factor in improving academic performance and interpersonal relationships with members of the academic community.^4^ ^,^ ^9^ The enhancement of soft skills is directly related to the development of emotional intelligence.^10^ High levels of emotional intelligence are also related to a mutual improvement in the dentist-patient relationship.^6^ ^,^ ^11^ Healthcare providers showed better working management strategies, more empathy, and also meeting patient expectations more often when they develop their emotional intelligence.^12^ Additionally, patients are less anxious, more adherent to treatment, and more faithful to dentists with higher levels of emotional intelligence.^6^

The present results demonstrated that 54,6% of orthodontists have not received any instructions on soft skills during their professional training. However, the soft skills were frequently used in their daily practice. These findings indicated that dental schools focus their efforts on the theoretical and technical training of residents in orthodontics.^1^ Recent efforts to broaden the debate on emotional abilities in universities were performed.^13^ However, there is still a worldwide deficit of attention to soft skills in the academic curriculum.^12^

Interestingly, residents demonstrated to access reliable research sources less frequently than young orthodontists. Self-guided education in adults is related to characteristics such as maturity, responsibility, and previous experiences.^13^ Consequently, residents in Orthodontics may still present some difficulties accessing reliable research sources, since they have not been exposed to similar circumstances with the same frequency. 

In addition, women reported to seek help from other professionals more often than men. These findings agree with the literature, in which female dentists tended to be more conservative in their treatments, refer patients more frequently, and use their communication skills more often and more effectively than men.^14^ ^,^ ^15^ One limitation of the present study was the sex distribution among the participants. A greater number of women answered the questionnaire, when compared to men (92 women and 37 men). This phenomenon may be associated with the current feminization trend in Dentistry. An increase in the active participation of women in Dentistry has been observed in the last decades.^14^ ^,^ ^15^ The increase in the proportion of women in Dentistry caused changes in working patterns and career satisfaction.^16^ Although the profession may shift toward less entrepreneurship to more urbanization, feminization in Dentistry is associated with more empathy, communicative and preventive strategies.^15^

Among all listed soft skills, ethics and communication were more frequently ranked as the most important ones. Although undergraduate curricula include theoretical instruction regarding the legal aspect of Dentistry, it is also important to provide students with clinical skills as well.^17^ Ethics can be considered a concern for orthodontists, since they often face ethical issues in their daily practice.^18^ It is important that these professionals use their communicative skills, informing properly the patient about treatment, procedures and factors that may interfere with the professional’s ability to obtain a more favorable prognosis.^19^ The patient must sign a dental service contract and informed consent form, agreeing with the treatment plan, and be aware of all possible incidents and limitations.^19^

Communication has been the most studied and mentioned soft skill in the literature.^20^ Understanding communication in its different forms is fundamental to dealing with the demands of patients and their anxieties.^20^ ^,^ ^21^ Effective communication is associated with lower levels of stress, better patient adherence and loyalty, and reduced professional negligence.^22^ On the other hand, lack of communication skills is associated with difficulties in the relationship with patients and with significant unemployment rates.^20^ ^,^ ^21^

Among all listed soft skills, information management and leadership were less frequently ranked as important skills. The analysis of the dentist-patient relationship and its effectiveness has been directed towards more empathic and less paternalistic bonds.^23^ Therefore, even if a professional hierarchy is necessary, there is a tendency towards an egalitarian relationship where the dentist is not placed as a leader, but as a partner during treatment, health improvement and quality of the patient’s life. However, residents should be better trained regarding leadership skills related to teamwork. When defining characteristics of financially successful orthodontists, leadership is mentioned for maximizing talents and teamwork, in order to provide exemplary patient care and attain increased levels of production.^24^

A recent resolution by the Brazilian Ministry of Education highlighted the importance of some of the soft skills for Dentistry graduate education.^25^ New curriculum guidelines were recommended, directing efforts towards development, especially in communication and leadership skills. Although leadership skill was highlighted by competent educational institutions, it was one of those considered least important by the participants of this research. In general, it is important to see the discourse of soft skills reaching political-educational dimensions around the world.

All research participants were from the same institution, and this is a limitation, since the use of soft skills may vary according to cultural and behavioral aspects.^2^ Due to the low response rate of the data collected by the research, the consequent results should be considered with caution, and not generalized, as the respondents represent only a portion of this population. However, it is important to highlight the originality and uniqueness of this first analysis for the orthodontic field. Further multicentric studies should be performed, with a larger sample and evaluating new generations of orthodontists. Evaluation of the influence of soft skills on patient collaboration with removable orthodontic appliances and oral hygiene should also be performed.

## CONCLUSION

Conclusions are representative of the sampled population within the presented context. Residents in Orthodontics and experienced orthodontists had not received any training regarding soft skills. Newly graduated orthodontists were more frequently able to search and access reliable sources of information than residents in Orthodontics. Women demonstrated seeking professional help more often than men. Ethics and communication were more frequently ranked as the most important skills in professional life. Information management and leadership were less frequently ranked as important skills. 
